# Brown-Vialetto-Van Laere syndrome

**DOI:** 10.22037/ijcn.v18i2.37314

**Published:** 2024-03-12

**Authors:** Shima IMANNEZHAD, Ehsan GHAYOOR KARIMIANI, Majid SEZAVAR, Gholam Reza KHADEMI, Maryam NASERI, Farah ASHRAFZADEH

**Affiliations:** 1 Department of Pediatrics, Faculty of Medicine, Mashhad University of Medical Sciences, Mashhad, Iran.; 2 Innovative Medical Research Center, Mashhad Branch, Islamic Azad University, Mashhad, Iran

**Keywords:** Brown-Vialetto-Van Laere Syndrome, Hearing loss, Bulbar palsy, Riboflavin

## Abstract

Brown-Vialetto-Van Laere syndrome (BVVLS) is a rare neurodegenerative disorder of childhood. According to the previous reports, it has various primary signs and symptoms. Because of the simple treatment with riboflavin supplementation, it is important to have suspicious to this disease and begin treatment even before genetic test confirm. We report a five-year-old girl with BVVLS that manifest with hearing problems, first. There was obvious improvement in her disease clinical signs with riboflavin supplementation treatment.

## Introduction

Brown-Vialetto-Van Laere syndrome (BVVLS) is a rare neurodegenerative disorder of childhood characterized by the most prevalent symptoms of bulbar palsy, respiratory failure, hearing loss, and facial weakness. Mutations in the SLC52A3, formerly C20orf54 gene (one of the three known riboflavin transporter genes which encode the intestinal (hRFT2) riboflavin transporter) has been proposed as a reason for BVVLS^1^. The prevalence of the BVVLS is very low, and few cases have been diagnosed worldwide. It is known as the same disease entity with Fazio-Londe syndrome (FLS) with the symptom of deafness 2. BVVLS is a very severe syndrome that affects the riboflavin-dependent motor neurons with rapid progression of the symptoms and even fatal outcomes; so, early diagnosis and treatment can improve the clinical signs and could have striking and often lifesaving effects. The proper diagnosis of the syndrome requires mutation analysis of the riboflavin transporter genes; however, immediate treatment with riboflavin supplementation is highly recommended without waiting for the genetic test results^3^. Despite the reported cases with diagnosed BVVLS and treatment of riboflavin supplementation, the exact pathophysiology of the disorder and the optimal dose and frequency of administration of the riboflavin needs to be studied more. Here, we report a diagnosis of BVVLS and treatment of the symptoms in a child following the onset of hearing disorder.

## Case Report

The patient was a five-year-old girl admitted to the neurology department of the Ghaem hospital complaining the hearing loss progressed in the last seven months. The early symptoms of hearing impairment were observed when she was three years old. The auditory brainstem response (ABR) test and the auditory steady-state response (ASSR) were abnormal and showed auditory neuropathy. So, hearing aids were used. The bilateral ptosis, facial paralysis, drooling, and drooping of the right eyelid and mouth corner were also observed during the last five months, which were not worsen at night.

Her medical documents showed no developmental delay. She was the first child of healthy parents; however, her parents had a consanguineous marriage, which increased the risk of inherited genetic disease of the child. Her weight was 21.5 Kg, height 114.5 cm and head circumference 53.5 cm. Electromyography (EMG) and nerve conduction velocity (NCV) tests were normal. Laboratory test of complete blood count, ESR, CRP, blood urea, creatinine, ALT, AST were within the normal range.Thyroid profile, serum levels of calcium, pyruvate, lactate, ammonia and CPK were normal. Her blood gases were within normal limits.

**Figure 1:**
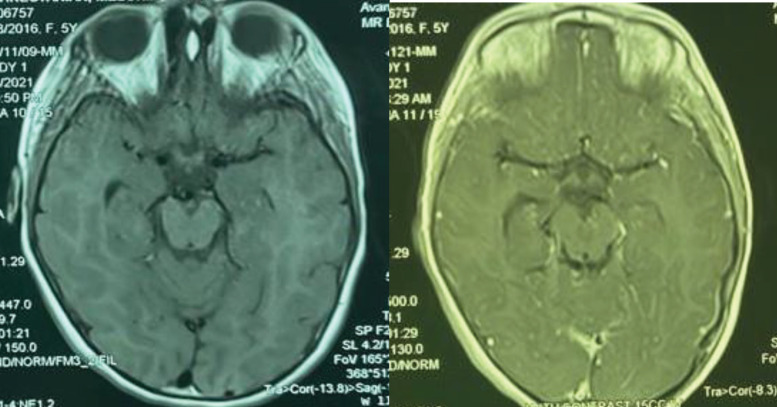
Brain MRI upon admission

Anti-MuSK Abs evaluated by radioimmunoprecipitation were negative. AChR binding antibody was upper limit of normal range. The patient did not respond to pyridostigmine with the doses 120 mg/day (divided in four parts). She underwent brain MRI ±GAD and it was normal (Figure 1). Cerebrospinal fluid (CSF) analysis obtained by a lumbar puncture after MRI, that was normal in view of sugar, protein, cytology and culture and, malignant cell was not found.

**Figure 2:**
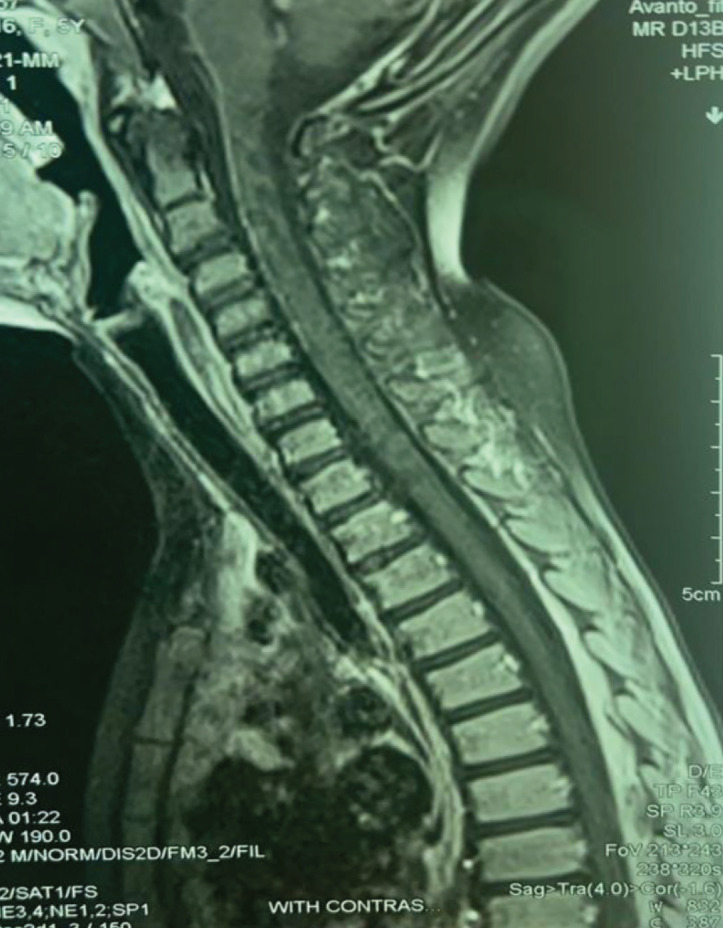
Spinal MRI upon addmision

Thoracic CT was normal. Thoracic and cervical MRI was also performed for the patient; the results didn’t show any pathology (Figure 2).

High-dose oral supplementation of riboflavin (vitamin B2) between 10-15 mg/kg/day was prescribed and gradually improvement in the symptoms and signs on clinical examination appeared.

Due to the suspicion of the underlying hereditary neurodegenerative syndrome, a genetic test of WES (Whole Exome Sequencing) was performed.

After genetic counseling, completing the relevant questionnaire and obtaining inform consent form, five ml peripheral whole blood was collected in an EDTA tube from available family members. DNA extracted by GeneAll Kit. DNA sample has been sent for whole exome sequencing or WES (Macrogen, Korea). After bioinformatic data analysis, we tend to followed our studies with Sanger sequencing to verify the candidate variant in patients and in parents.

Co-Segregation analysis confirmed that the patient was mutated homozygous for the c.1036C>T (p.Pro346Ser) variant of the SLC52A3 gene. Her parents were relative and were both heterozygous carriers for this variant (Table1). The associated disease with mutations in the SLC52A3 gene was diagnosed as Brown-Vialetto-Van Laere syndrome.

**Table 1:** Whole exome sequencing test results

Relation	Zygosity	Gene: variant	Classification	Associated disease
Child (affected)	Mutated Homozygous	SLC52A3: NM_033409.4 c.1036C>T (p.Pro346Ser)	Class 3. Variant of uncertain clinical significance	Brown-Vialetto-Van Laere syndrome disease (211530) Autosomal Recessive
Mother	Heterozygous			
Father	Heterozygous			

## Discussion

We describe the case of a five-year-old child with progressive hearing loss, bulbar palsy, drooling, and bilateral ptosis. The mentioned clinical presentations increased the possibility of riboflavin transporter deficiency and the diagnosis of the BVVLS caused by the mutation in the SLC52A3 gene. The patient showed a substantial clinical improvement following immediate treatment with high dose oral riboflavin supplementation without waiting for the result of the molecular genetic test.

In addition to the clinical symptoms of the affected child, she was born to a consanguineous parent that also suspected the autosomal recessive inheritance and the diagnosis of the BVVLS. The differential diagnosis of BVVLS was consequently confirmed by mutation analysis. In agreement with previous studies, we found the homozygous mutation of c.1036C>T (p.Pro346Ser) in the SLC52A3 gene as the possible etiology of the BVVLS and riboflavin transporter deficiency 4, 5.

Similar cases with our patient have been also reported with bulbar palsies, sensorineural impairment, respiratory distress, and most of them were born to consanguineous families (the mean age of onset is eight years old). The critical role of defective riboflavin transport in Brown-Vialetto-Van Laere syndrome as infrequent motor neuron diseases of pediatric age group is confirmed in all the similar studies 1, 4, 6-8.

In a similar report, two siblings with progressive muscle weakness and paralysis of the diaphragm revealed homozygous mutation of SLC52A3 gene that encodes intestinal riboflavin transporter (hRFVT-3), and BVVL syndrome with autosomal recessive inheritance was diagnosed. Supplementation of riboflavin proved a lifesaving treatment in these cases 9. Similar to our finding, high dose riboflavin resulted in rapid clinical improvement and has been proposed as a potential treatment for BVVLS 9.

So, improvement of the clinical signs of BVVLS following high dose riboflavin supplementation supports the hypothesis that SLC52A3 gene mutation leads to decreased plasma riboflavin levels and is related to deficiency of riboflavin 10. In a recent case report, BVVLS was diagnosed in siblings following genetic examination. They had different phenotypes and onsets of symptoms; however, both showed lower motor neuron facial nerve palsy.

The differential diagnosis in the child presenting with progressive bulbar paralysis includes structural lesions of the brainstem, muscle diseases and disorders of neuromuscular transmission. Potentially treatable conditions such as myasthenia gravis, disorders of the cranial nerves and Miller Fisher’s syndrome should be excluded. Imaging is needed to exclude a structural abnormality in the brainstem or other degenerative diseases; for example, Alexander’s disease can occasionally present with bulbar paresis.

According to this report, in children with progressive bulbar palsy lower motor neuron facial palsy and hearing loss the diagnosis of BVVLS should be considered 4.

## In conclusion

Riboflavin supplementation is a simple and harmless therapy that should be immediately applied for children suspected for Brown-Vialetto-Van Laere syndrome even before genetically proved mutation.

## Author’s Contribution

Farah Ashrafzadeh: Responsible for the study design, collection and editing the manuscript.

Shima Iman Nezhad, Majid Sezavar, Gholam Reza Khademi, Maryam Naseri: Collecting data, writing the first draft of this manuscript.

Ehsan Ghayor Karimiani: have done the genetic study

## Conflict of Interest

None declared

## References

[1] Hossain MA, Obaid A, Rifai M, Alem H, Hazwani T, Al Shehri A, Alfadhel M, Eto Y, Eyaid W (2017). Early onset of Fazio-Londe syndrome: the first case report from the Arabian Peninsula. Hum Genome Var..

[2] Dipti S, Childs AM, Livingston JH, Aggarwal AK, Miller M, Williams C, Crow YJ (2005 ). Brown-Vialetto-Van Laere syndrome; variability in age at onset and disease progression highlighting the phenotypic overlap with Fazio-Londe disease. Brain Dev.

[3] Bosch AM, Stroek K, Abeling NG, Waterham HR, Ijlst L, Wanders RJ (2012 ). The Brown-Vialetto-Van Laere and Fazio Londe syndrome revisited: natural history, genetics, treatment and future perspectives. Orphanet J Rare Dis.

[4] Gowda VK, Udhayabanu T, Varalakshmi P, Srinivasan VM, Ashokkumar B (2018 ). Fazio-Londe syndrome in siblings from India with different phenotypes. Brain Dev.

[5] Cali E, Dominik N, Manole A, Houlden H, Adam MP (2015). Riboflavin Transporter Deficiency.

[6] Johnson JO, Gibbs JR, Megarbane A, Urtizberea JA, Hernandez DG, Foley AR, Arepalli S, Pandraud A, Simón-Sánchez J, Clayton P, Reilly MM, Muntoni F, Abramzon Y, Houlden H, Singleton AB (2012). Exome sequencing reveals riboflavin transporter mutations as a cause of motor neuron disease. Brain.

[7] Udhayabanu T, Subramanian VS, Teafatiller T, Gowda VK, Raghavan VS, Varalakshmi P, Said HM, Ashokkumar B (2016). SLC52A2 [p P141T] and SLC52A3 [p N21S] causing Brown-Vialetto-Van Laere Syndrome in an Indian patient: First genetically proven case with mutations in two riboflavin transporters. Clin Chim Acta.

[8] Varadarajan P, Thayanathi V, Pauline LC (2015 ). Fazio Londe syndrome: A treatable disorder. Ann Indian Acad Neurol.

[9] Bosch AM, Abeling NG, Ijlst L, Knoester H, van der Pol WL, Stroomer AE, Wanders RJ, Visser G, Wijburg FA, Duran M, Waterham HR (2011 ). Brown-Vialetto-Van Laere and Fazio Londe syndrome is associated with a riboflavin transporter defect mimicking mild MADD: a new inborn error of metabolism with potential treatment. J Inherit Metab Dis.

[10] Haack TB, Makowski C, Yao Y, Graf E, Hempel M, Wieland T, Tauer U, Ahting U, Mayr JA, Freisinger P, Yoshimatsu H, Inui K, Strom TM, Meitinger T, Yonezawa A, Prokisch H (2012). Impaired riboflavin transport due to missense mutations in SLC52A2 causes Brown-Vialetto-Van Laere syndrome. J Inherit Metab Dis.

